# Biographical

**Published:** 1896-05

**Authors:** 


### Biographical.

Died, at his home in Indianapolis, April 24th, of disease of the heart, Dr. Phineas G. C. Hunt, aged sixty-eight years. Dr. Hunt was born in Champaign County, Ohio, in 1827. In 1833 he, with his mother, who was a widow, with her other children, removed to Indiana. He received a common school education in Wayne County, near Richmond, Ind. His winters were devoted to school work, and during the other portions of the year he was engaged in farm labor. At about the age of nineteen years he began to consider the question of a life occupation. Having a brother, David P. Hunt, then practicing dentistry in Indianapolis, he made a visit to his brother, which resulted in his becoming a student in dentistry in 1846. Two years later his brother died, and Dr. Hunt found the duties and responsibilities of a full practice suddenly upon his hands, while yet scarcely more than a boy. He thus began with a full and independent practice at the age of twenty-one years. The professional career of Dr. Hunt extended over forty-five years in one community, and was one of almost unparalleled success. Throughout his professional life he was recognized as a leader in the dental profession in Indiana, and one who scrupulously maintained the dignity of his calling. He was always recognized as being well up with the times, often reaching far in advance of his co-laborers. Dr. Hunt was one of the first dentists in the West to cast aside the prejudices and jealousies once so characteristic of the profession of that day, and to throw open his doors to his brethren, imparting to all seekers the knowledge with which he was so well equipped. He was a frequent contributor to the dental journals. He also originated many ideas in practice and devised a number of instruments and appliances in connection with dentistry. He was one of the first to illustrate that new teeth could be extracted and afterwards replaced in the jaw so that they would become fixed and useful after the gums had been properly treated. He was an enthusiastic experimenter. A little incident will fully illustrate this: One day in the '70s he called a number of his friends to his home, where the first thing he did was to direct them to the poultry-yard, where he had on exhibition some half dozen roosters, each one of which had a human tooth firmly grown in its crest, thus illustrating the principle which has elicited so much discussion since that time. He was a graduate of the Ohio College of Dental Surgery; he also received the title of M.D. from an Indiana medical college. He was a member of many dental societies, and was a member of the Board of Dental Examiners of the State of Indiana, of which board he was President many years. The following action was had at a meeting of the dentists of Indianapolis the day after his death:

"A meeting of local dentists was held at the office of Dr. T. S. Hacker, April 25th, to take action with reference to the death of Dr. Hunt, who was the oldest practicing dentist in this city. There were present the following: T. S. Hacker, Merit Wells, Alex. Jameson, William S. Rawls, E. E. Reese, Elmer Smythe, A. J. Morris, Willard Gates, Dr. George, Robert T. Oliver, D. H. Oliver, David B. House, J. B. Morrison, Frank A. Hamilton, J. Q. Byran, Maurice Raschig and J. E. Cravens.

"The following preamble and resolutions were adopted:

"WHEREAS, The death of Dr. P. G. C. Hunt of this city has removed from the ranks of active dentists one of its foremost and most honored members, therefore, be it

"*Resolved*, That in the death of Dr. Hunt the dental profession of this city and State has sustained irreparable loss. Dr. Hunt in the highest sense represented the spirit of progress in his chosen profession. His death takes from us the best representative of the self-made practitioner. Dr. Hunt early made his influence felt in the national councils of dentistry and his ideas are to be found embalmed in the books of to-day in the schools of dentistry. The history of American dentistry can not be written without contributing to his praise.

"*Resolved*, That a copy of these resolutions and note of this meeting be transmitted to the bereaved family of Dr. Hunt, and that the daily papers be requested to publish the same.

"A beautiful floral tribute was ordered to be placed upon the casket. It will contain the inscription, 'From the Dentists of Indianapolis.' It was decided to attend the funeral in a body and to extend invitations to all dentists of the city and vicinity to join them."

